# Self-Reported Sleep Quality Modulates Amygdala Resting-State Functional Connectivity in Anxiety and Depression

**DOI:** 10.3389/fpsyt.2018.00220

**Published:** 2018-05-29

**Authors:** Heide Klumpp, Bobak Hosseini, K. Luan Phan

**Affiliations:** ^1^Mood and Anxiety Disorders Research Program, Department of Psychiatry, University of Illinois at Chicago, Chicago, IL, United States; ^2^Department of Psychology, University of Illinois at Chicago, Chicago, IL, United States; ^3^Mental Health Service, Jesse Brown VA Medical Center, Chicago, IL, United States

**Keywords:** fMRI rest, anxiety, depression, sleep, insomnia, neuroimaging

## Abstract

Sufficient sleep plays an important role in neurocognitive function, yet, problematic sleep is ubiquitous in the general population. It is also frequently predictive of, and concurrent with, internalizing psychopathologies (IPs) such as anxiety and depression suggesting sleep quality is dimensional and transdiagnostic. Along with problematic sleep, IPs are characterized by negative affectivity, therefore, prominent neurobiological models of internalizing conditions involve the amygdala, a region central to emotion. In resting-state studies (independent of sleep considerations), abnormalities in amygdala-frontal functional connectivity are commonly observed suggesting emotion dysregulation may contribute to clinically-relevant phenotypes. In a separate line of research, studies of sleep deprivation, and insomnia disorder suggest sleep loss may alter amygdala-frontal connectivity. Taken together, findings point to shared neurobiology between sleep and emotion systems, however, the impact of sleep quality on the amygdala circuit in anxiety or depression is unclear. Therefore, we evaluated variance in naturalistic sleep quality on amygdala-based circuity in individuals with and without psychiatric illness. Resting-state fMRI data was collected in 87 un-medicated, treatment-seeking adults diagnosed with a primary anxiety disorder (*n* = 68) or primary depressive disorder (*n* = 19) in addition to healthy individuals (*n* = 40). Regression analysis was conducted with bilateral anatomical amygdala as seed regions and self-reported sleep quality was indexed with a validated self-report measure, the Pittsburgh Sleep Quality Index (PSQI). *Post-hoc* analysis was performed to evaluate whether diagnostic status (primary anxiety, primary depression, healthy) significantly explained functional connectivity results. Whole-brain regression analysis, controlling for anxiety and depression symptoms, revealed worse sleep quality (i.e., higher PSQI total scores) predicted increased left amygdala-subgenual anterior cingulate functional connectivity and reduced connectivity with posterior cerebellar lobe and superior temporal gyrus. For right amygdala, increased coupling with postcentral gyrus corresponded with worse sleep. *Post-hoc* analysis did not detect a significant relationship between diagnostic status and whole-brain findings. Results expand on previous studies and indicate variance in sleep quality tracks brain pathways involved in cognitive-emotion functions implicated in the neurobiology of IPs that may extend to individuals at risk for clinical anxiety or depression. Altogether, the clinical relevance of identifying phenotypes to improve our understanding of psychopathology may be improved by incorporating sleep quality.

## Introduction

Sufficient sleep is critical for optimal brain function (1, 2) yet problematic sleep such as difficulty falling asleep, staying asleep, waking up too early, and other symptoms of insomnia is prevalent in the general population [e.g., about 30% of adults; (3, 4)] and has a negative impact on mood, cognitive functions, and health placing individuals at increased risk for mortality [e.g., (5, 6)]. Problematic sleep indicative of insomnia is marked by hyper-arousal [e.g., elevated autonomic and physiological activity; (7–9)] and poor sleep is a risk factor in the development and maintenance of mood disorders (10–12). Moreover, common internalizing psychopathologies (IPs) such as major depression, generalized anxiety disorder, social anxiety disorder, and panic disorder (13, 14) are highly comorbid with problematic sleep. For these anxiety disorders, 60–90% report sleep disturbances and for major depression, estimates are 50–83% (15–19). Notably, these IPs are also frequently comorbid with each other. For example, most individuals with major depression will have experienced a concurrent anxiety disorder [e.g., 59%; (20)] and up to 90% of individuals with an anxiety disorder will have had comorbid depression (21) indicating certain shared neural substrates across IPs.

Given strong links between poor sleep and anxiety and depression, variance in sleep quality would be expected to interact with neural pathways implicated in IPs. Yet, the impact of naturalistic sleep in the brain pathophysiology of IPs has been a neglected area of study potentially due to the general view that problematic sleep is a “nuisance” variable (e.g., secondary to primary dysfunction). However, this view is changing given evidence of shared neurobiology between sleep and emotion systems suggesting abnormalities in brain circuits may reflect dysfunction that converge on bidirectional sleep-emotion mechanisms (2, 22, 23).

A hallmark of IPs is negative affectivity (24) and influential neurobiological models of IPs involve the amygdala, a central structure in the neural circuitry of emotion (25, 26). Independent of sleep considerations, individuals with anxiety or depression frequently exhibit excessive amygdala reactivity to salient, negative stimuli compared to healthy participants (27, 28), and though less well studied, problematic sleep is also associated with an amplified amygdala response to relevant, negative stimuli (29) further supporting close ties between IPs and sleep loss. The amygdala has extensive connections in a distributed cortical–subcortical network (30–33), therefore, examining sleep quality in an amygdala-based circuit may advance the identification of phenotypes that cut across conventional diagnostic boundaries (34, 35).

Resting-state functional connectivity (rsFC) is an effective, efficient method for evaluating the relationship of large-scale intrinsic spontaneous brain activity between regions thought to reflect direct and indirect anatomical connections (36–39). Highlighting the widespread functional cortical-subcortical interactions with amygdala, Roy et al. (40) demonstrated spontaneous amygdala activity in healthy individuals is positively predicted by regions involved in evaluative functions, emotion processing, and cognitive-emotion interactions [e.g., anterior cingulate cortex, medial prefrontal cortex, insula, striatum, thalamus; (31, 32, 41)], and negatively coupled with regions associated with internally-directed cognition and effortful emotion regulation [e.g., middle frontal gyrus, posterior cingulate gyrus, precuneus; (42–45)]. In case-controlled rsFC studies with amygdala as the seed region, data show aberrant (hypo- or hyper-connectivity) in cortical and subcortical areas in insomnia, depression, and anxiety. For example, individuals with insomnia disorder (without concurrent psychiatric illness) exhibit altered rsFC between amygdala and the inferior frontal gyrus, subcortical structures (e.g., caudate, thalamus), temporal, parietal, and occipital regions relative to healthy controls (46). Also, limited research comparing insomnia disorder to an IP reported relatively more amygdala-rostral anterior cingulate cortex (ACC) rsFC in primary insomnia compared to generalized anxiety disorder (47). Though findings suggest a compensatory response in insomnia, both groups exhibited less amygdala-rostral ACC coupling than good sleepers signifying diminished top-down control of emotion regulation in these disorders (47). Similar dysfunction has been observed in IPs.

Broadly, anomalous amygdala-related rsFC in circuits that encompass emotion, cognition, emotion regulation, and sensorimotor processes are observed in depression and anxiety disorders. Although results across studies are inconsistent as to the pattern of aberrant connectivity and specific regions (48–55), reduced amygdala-frontal connectivity involving the medial prefrontal cortex (PFC) or ACC has been frequently reported. For example, compared to healthy participants, socially anxious individuals exhibit decreased amygdala-rostral ACC rsFC (55); though see (48); high trait (vs. low trait) anxious individuals, reduced amygdala-ventral medial (VMPFC) rsFC (51); adolescents with generalized anxiety disorder, decreased amygdala-dorsolateral PFC rsFC (52); adolescents with major depression, reduced amygdala-medial PFC rsFC (53); though see (49); and clinically anxious youth, decreased amygdala-VMPFC rsFC (50). While methodological differences across studies (e.g., participant sample including comorbidity, analytic approach) preclude direct comparison between studies, findings generally point to reduced amygdala-frontal rsFC in IPs. However, the extent to which problematic sleep in IPs modulates intrinsic connectivity in the amygdala circuit is unclear.

Therefore, the primary objective in the current study was to expand on the literature by examining the relationship between sleep quality and amygdala-based rsFC in individuals with and without clinical anxiety or depression. Since insufficient sleep is ubiquitous (3, 4), healthy individuals were expected to vary in sleep quality as well. We hypothesized individual differences in subjective sleep would converge on an amygdala-frontal circuit and that less rsFC in this circuit would be predicted by worse sleep across participants.

## Methods

### Participants

As part of a larger study, 87 treatment-seeking adults diagnosed with a primary anxiety disorder (*n* = 68) or primary depressive disorder (*n* = 19) participated. Healthy controls (HC; *n* = 40) completed the same tasks at the same time points as patients. All 127 participants completed a consent form approved by the local Institutional Review Board at the University of Illinois at Chicago. All participants met with a master's-level clinician or PhD/MD assessor, who performed the Structured Clinical Interview for DSM-5 (56) and other clinician-administered measures. Comorbidity was permitted, therefore, the Hamilton Depression Rating Scale [“HAM-D”; (57)] and Hamilton Anxiety Rating Scale [“HAM-A”; (58)] assessed symptom severity across disorders. All measures, including the Pittsburgh Sleep Quality Index (PSQI) (described below), were collected within a week of the fMRI scan and before receiving pharmacotherapy or psychotherapy. All participants tested negative on a urine toxicology screen before the scan.

All participants in this study were free from psychotropic medication and free from major medical and neurological illness as confirmed by a Board Certified physician. Exclusion criteria for healthy participants included a current or past Axis I disorder. Exclusion criteria for all participants were less than 18 or more than 65 years of age, contraindications to magnetic resonance imaging (e.g., pregnancy, ferrous objects), current substance dependence (within 6 months of the study), history of other major psychiatric illness (e.g., bipolar disorder, psychotic disorders), or current cognitive dysfunction (e.g., traumatic brain injury, pervasive developmental disorder). All participants were compensated for their time and all procedures complied with the Helsinki Declaration.

### Subjective measure of sleep quality

The PSQI is a 19-item self-report questionnaire that assesses sleep disturbance over the past month (59). The total score comprises 7 component scores: subjective sleep quality, sleep latency, sleep duration, habitual sleep efficiency, sleep disturbances, use of sleeping medication, and daytime dysfunction. Scores range from 0 to 21 and higher scores represent worse sleep (59).

### Resting-state fMRI

Padding with foam cushions was used to reduce head movement. The participants were instructed to fixate on a crosshair centrally displayed on the blank gray screen, relax, and let their mind wander without falling asleep for 8 min.

### fMRI data acquisition and preprocessing

Scanning was conducted on a 3 Tesla GE Discovery System (General Electric Healthcare; Waukesha, WI) with an 8-channel head coil. Functional data were acquired using gradient-echo echo planar imaging (EPI) sequence with the following parameters: TR = 2 s, TE = minFull [~25 ms], flip angle = 90°, FOV = 22 × 22 cm^2^, acquisition matrix 64 × 64, 3-mm slice thickness, 44 axial slices, 180 volumes per run. For anatomical localization, a high-resolution, T1-weighted volumetric anatomical scan was acquired.

Connectivity analyses were performed using the Functional Connectivity (CONN) toolbox, which employs procedures from the Statistical Parametric Mapping software (SPM8; Wellcome Trust Centre for Neuroimaging, London, UK). Four initial volumes from each resting-state run were discarded to allow for T1 equilibration effects. Images were realigned to correct for motion, corrected for errors in slice timing, spatially transformed to standard MNI space using the functional template provided with SPM8, resampled to 2-mm voxels, and smoothed with an 8-mm FWHM Gaussian kernel prior to statistical analysis. All participants had no movement greater than 2-mm translation or 2 degrees rotation across the run. Effects of nuisance variables (global, white matter and CSF signals and movement parameters) were reduced following the CompCor strategy (60); data were band-pass filtered to 0.01–0.09 Hz.

In the seed-based analyses, temporal correlations of the resting-state BOLD signal time series were examined between the left and right amygdala regions anatomically derived from the Automated Anatomical Labeling (AAL) toolbox (61) and the rest of the brain. During second-level processing, PSQI total scores were regressed as a covariate of interest. To control for symptoms, HAM-A and HAM-D total scores were entered as a covariate of no interest. Specifically, due to the high concordance between these measures (*r* = 0.81, *p* < 0.001), the summation of HAM-A and HAM-D was used to control for symptom severity.

Following recent guidelines in response to concerns about false positives resulting from lenient significance thresholds (62, 63), whole-brain activity was considered significant if it exceeded adjustment for multiple comparisons across the entire brain (e.g., a whole-brain gray matter mask [volume = 1,459,304 mm^3^]) as determined via simulation using the 3dClustSim utility (10,000 iterations; updated and “bug-free” on December 2015; [https://afni.nimh.nih.gov/pub/dist/doc/program_help/3dClustSim.html]; 64). Significance at α < 0.05 and a voxel threshold of *p* < 0.001 yielded a minimum cluster size of 26 voxels (volume = 208 mm^3^) for the regression analysis.

For significant whole-brain findings, parameter estimates of peak activation (β weights, arbitrary units [a.u.]) were extracted from spherical (10-mm diameter) regions of interest (ROI) from each participant and submitted to Pearson's correlations and scatter plots in the Statistical Package for the Social Sciences (SPSS, Version 22) to illustrate the magnitude and direction of significant effects established at the whole-brain level. To evaluate whether findings were driven by a particular diagnostic group, significant regression results were submitted to *post-hoc* linear regression analysis in SPSS (enter method) where the neural activity extracted with the spherical ROI was the dependent variable and diagnostic status (dummy coded for primary anxiety, primary depression, and healthy control) was the independent variable (Model 1). To evaluate whether any component(s) of the PSQI significantly predicted whole-brain findings, the same regression analysis was performed (enter method) though the independent variable consisted of the seven PSQI components (Model 2).

## Results

### Participants

Eighty-eight of the 127 participants were female (69.3%). The average age in years was 25.6 ± 7.5 and the average education level in years was 15.8 ± 2.8. PSQI total scores ranged from 0 to 20 and the average was 6.6 ± 4.3. A PSQI total score > 5 denotes problematic sleep (sensitivity 89.6%, specificity 86.5%) (59), therefore, a portion of participants had substantial sleep difficulties. Regarding race/ethnicity, 60.6% self-identified as Caucasian, 21.3% as Asian or Pacific Islander, 9.4% as African American, 6.4% as other or unknown, and 18.9% as Hispanic or Latino. See Table 1 for demographic characteristics and Table 2 for all concurrent disorders.

**Table 1:** Demographic and clinical characteristics.

	**Patients (*****N*** = **87)**		**Heathy participants (*****N*** = **40)**	
	***M*** **(SD)**		***M*** **(SD)**	
**MEASURES**				
HAM-A	14.2 (6.5)		1.0 (1.4)	
HAM-D	9.9 (5.0)		0.7 (1.0)	
Age	26.0 (7.5)		24.6 (7.5)	
**SELF-REPORTED SLEEP QUALITY**				
PSQI	8.1 (4.2)		3.5 (3.0)	
	***N***	**%**	***N***	**%**
**RACE/ETHNICITY**				
Caucasian	57	66.5	20	50.0
Asian	18	19.5	10	25.0
African American	6	6.89	6	15.0
American Indian or Alaskan Native or other/unknown	6	6.89	4	10.0
**GENDER**				
Male	25	28.7	14	35.0
Female	62	71.2	26	65.0

*HAM-A, Hamilton Anxiety Rating Scale; HAM-D, Hamilton Depression Rating Scale; PSQI, Pittsburgh Sleep Quality Index*.

**Table 2:** Distribution of primary diagnosis and comorbidity.

	***N***	**%**
**PRIMARY DIAGNOSIS**		
Social anxiety disorder	35	40.2
Generalized anxiety disorder	29	33.3
Major depressive disorder	18	20.7
Panic disorder	4	4.60
Persistent depressive disorder	1	1.15
**COMORBIDITY**		
Social anxiety disorder	23	26.4
Major depressive disorder	12	13.8
Generalized anxiety disorder	11	12.6
Specific phobia	9	10.3
Persistent depressive disorder	5	5.75
Panic disorder	4	4.60
Posttraumatic stress disorder	4	4.60
Eating disorder	1	1.15
Two or more concurrent diagnoses	11	12.6

### Whole-brain regression: left amygdala

**Figure 1:**
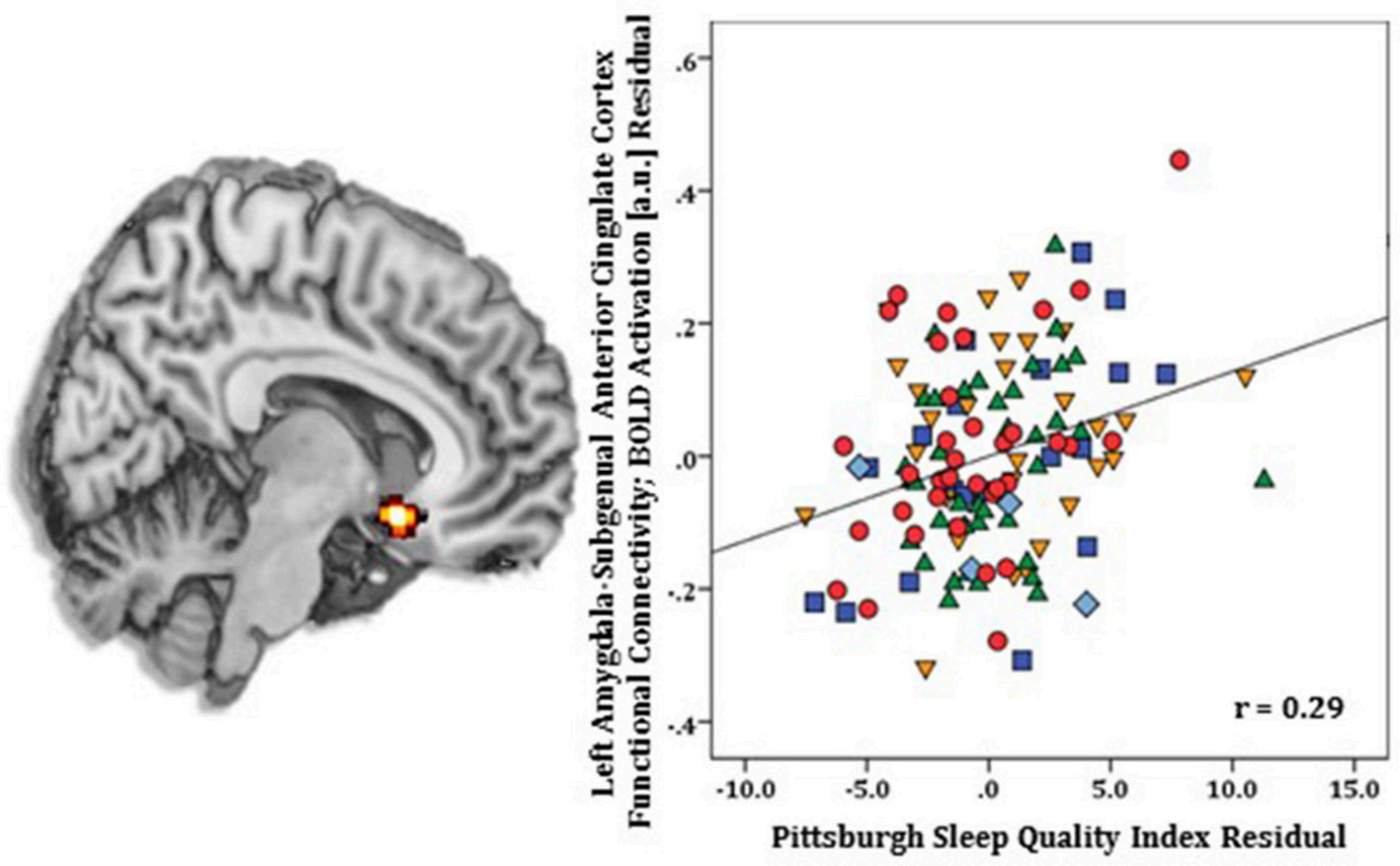
Whole-brain analysis of covariance with sleep quality, indexed with the Pittsburgh Sleep Quality Index (PSQI), showing amygdala-subgenual anterior cingulate cortex parameter estimates of functional connectivity, controlling for symptom severity, on a statistical t-map at *p* < 0.005 **(Left)**. Scatter plot of the regression analyses depicting extracted parameter estimates of functional connectivity between amygdala and subgenual anterior cingulate cortex, controlling for symptom severity, showing greater connectivity is associated with higher PSQI total scores **(Right)**. Green triangle, healthy participants; dark blue square, depression; orange inverse triangle, generalized anxiety disorder; light blue diamond, panic disorder; red circle, social anxiety disorder.

**Figure 2:**
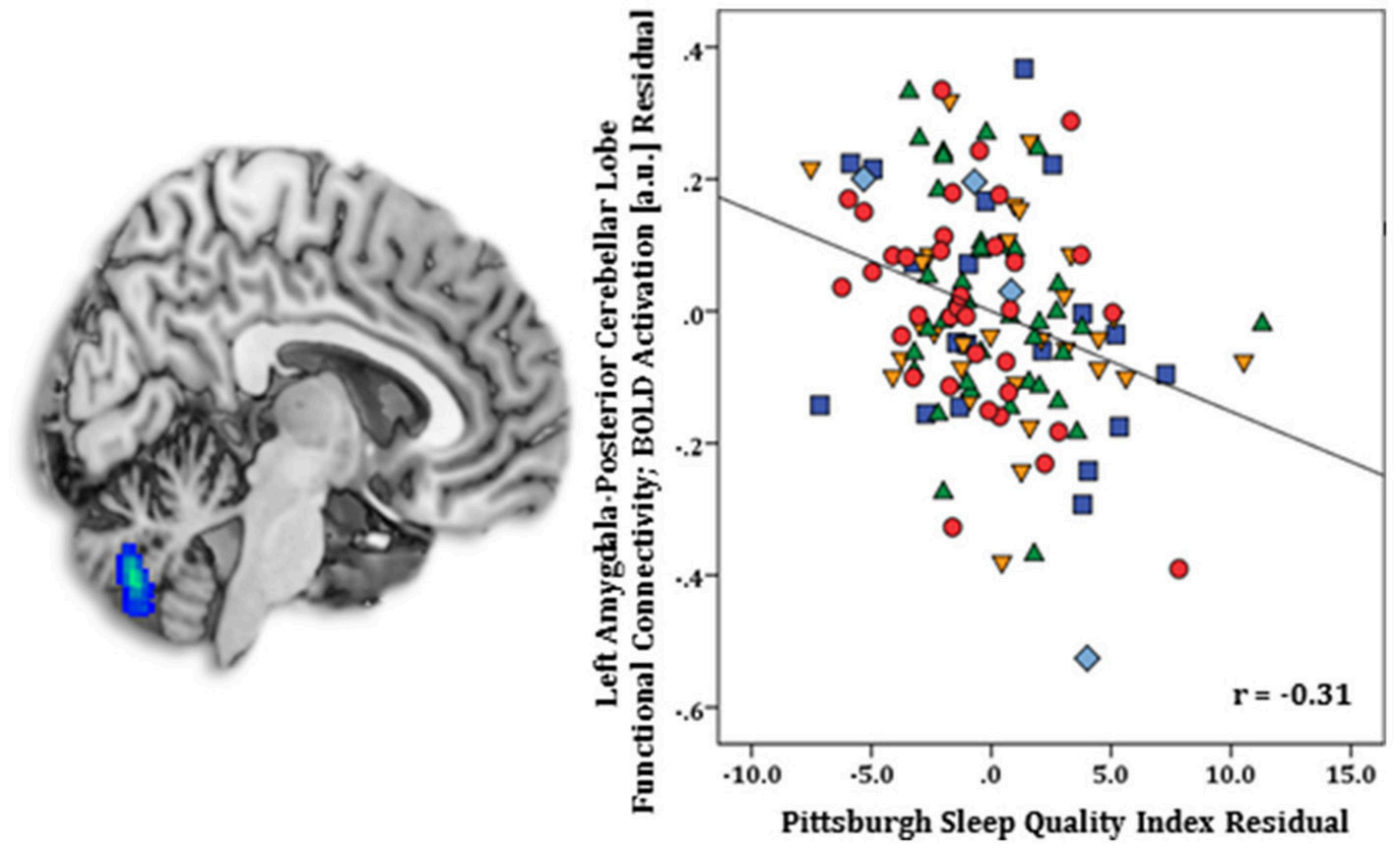
Whole-brain analysis of covariance with sleep quality, assessed with the Pittsburgh Sleep Quality Index (PSQI), showing amygdala-posterior cerebellar lobe parameter estimates of functional connectivity, controlling for symptom severity, on a statistical t-map at *p* < 0.005 **(Left)**. Scatter plot of the regression analyses depicting extracted parameter estimates of amygdala-posterior cerebellar lobe functional connectivity, controlling for symptom severity, illustrating greater connectivity corresponds with lower PSQI total scores **(Right)**. Green triangle, healthy participants; dark blue square, depression; orange inverse triangle, generalized anxiety disorder; light blue diamond, panic disorder; red circle, social anxiety disorder.

**Figure 3:**
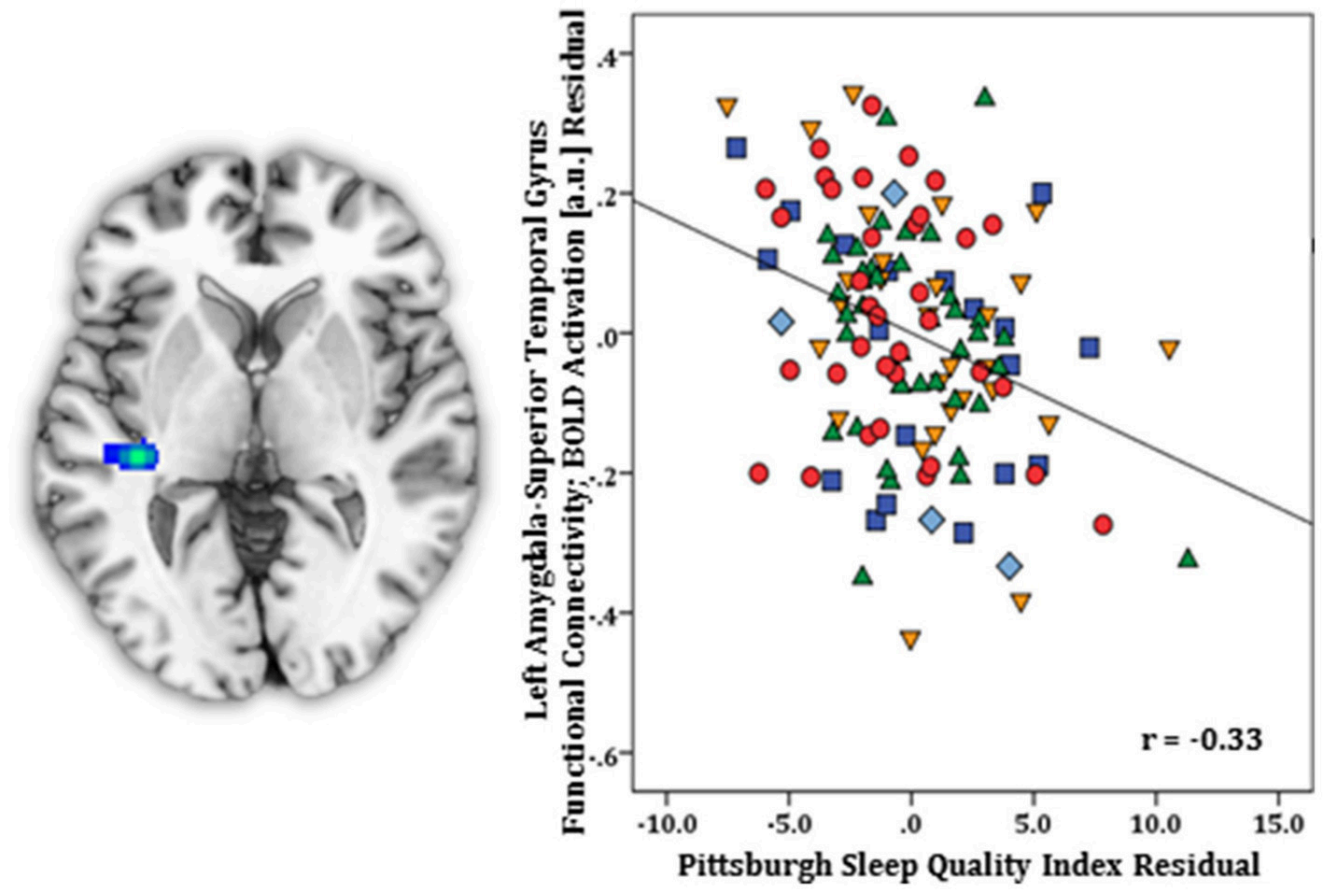
Whole-brain analysis of covariance with sleep quality, examined with the Pittsburgh Sleep Quality Index (PSQI), revealing amygdala-superior temporal gyrus parameter estimates of functional connectivity, controlling for symptom severity, displayed on a statistical t-map at *p* < 0.005 **(Left)**. Scatter plot of the regression analyses depicting extracted parameter estimates of amygdala-superior temporal gyrus functional connectivity, controlling for symptom severity, demonstrating greater connectivity is linked with lower PSQI total scores **(Right)**. Green triangle, healthy participants; dark blue square, depression; orange inverse triangle, generalized anxiety disorder; light blue diamond, panic disorder; red circle, social anxiety disorder.

Regression results, controlling for symptom severity, revealed higher PSQI total scores corresponded with increased connectivity to the right subgenual ACC [(8, 14, −12) *z* = 3.54, *k* = 28, volume = 224 mm^3^; *p* < 0.05 corrected] (Figure 1). In contrast, a negative connectivity pattern was detected for right posterior cerebellar lobe [(10, −70, −42) *z* = 3.75, *k* = 89, volume = 712 mm^3^; *p* < 0.05 corrected] (Figure 2) and left superior temporal gyrus [(−38, −26, 4) *z* = 4.04, *k* = 74, volume = 592 mm^3^; *p* < 0.05 corrected] (Figure 3).

In SPSS, *post-hoc* regression analysis showed diagnostic status did not predict connectivity results (lowest *p* = 0.16) (Model 1). Also, PSQI component scores were not significantly related to amygdala-subgenual ACC (*p* = 0.09) or amygdala-cerebellar (*p* = 0.10) functional connectivity (Model 2). However, amygdala-superior temporal gyrus coupling was predicted by the one-item overall sleep quality component [*R*^2^ = 0.16, *F*_(7, 119)_ = 3.20, *p* < 0.004] (B = −0.051, *t* = −1.98, *p* < 0.05) but no other components (all *p's* > 0.05).

### Whole-brain regression: right amygdala

**Figure 4:**
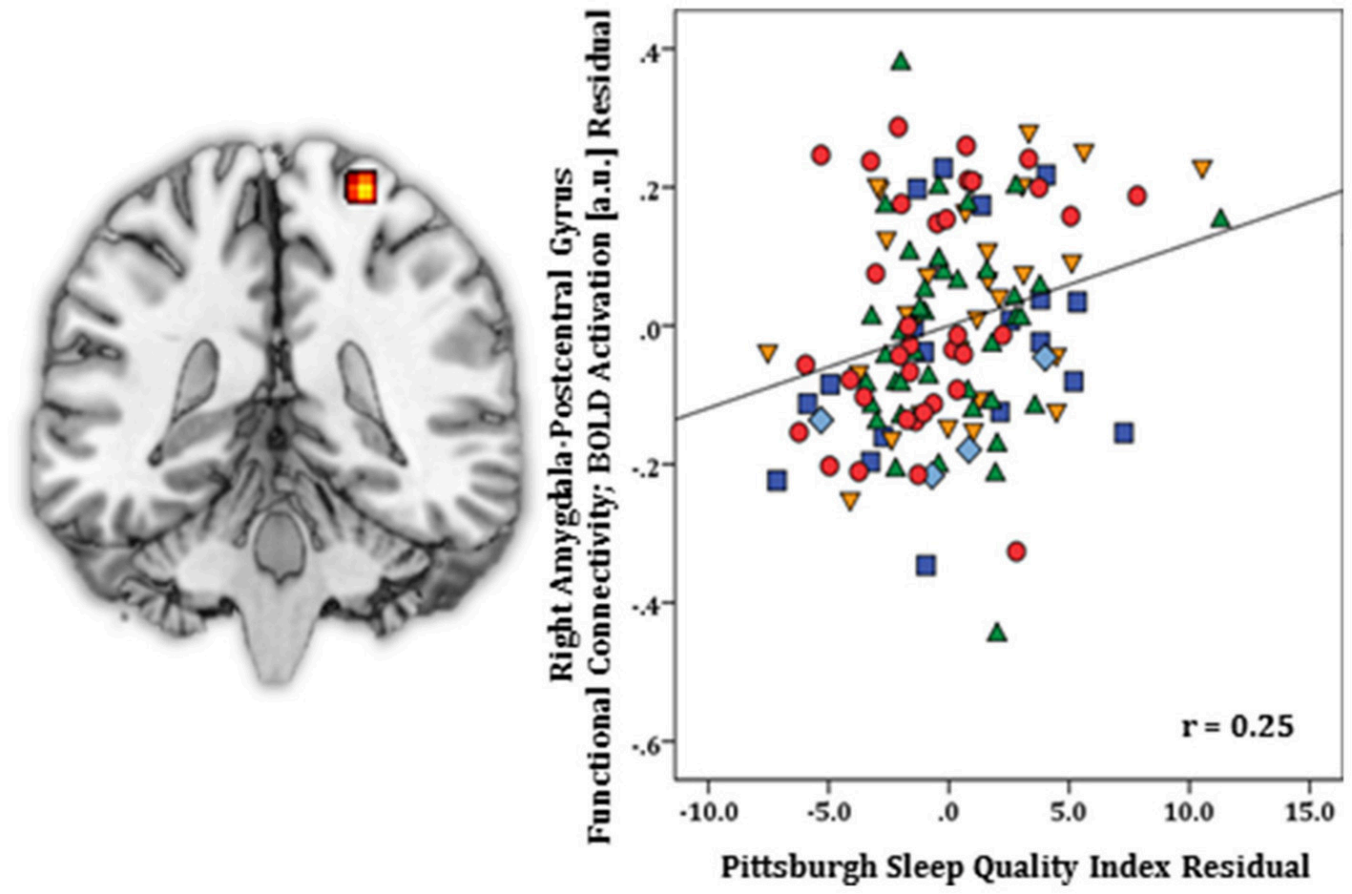
Whole-brain analysis of covariance with sleep quality, indexed with the Pittsburgh Sleep Quality Index (PSQI), showing amygdala-postcentral gyrus parameter estimates of functional connectivity, controlling for symptom severity, on a statistical t-map at *p* < 0.005 **(Left)**. Scatter plot of the regression analyses depicting extracted parameter estimates of functional connectivity between amygdala and postcentral gyrus, controlling for symptom severity, showing greater connectivity is associated with higher PSQI total scores **(Right)**. Green triangle, healthy participants; dark blue square, depression; orange inverse triangle, generalized anxiety disorder; light blue diamond, panic disorder; red circle, social anxiety disorder.

Regression results, controlling for symptom severity, showed increased functional connectivity to the right postcentral gyrus [(24, −34, 70) *z* = 3.64, *k* = 29, volume = 232 mm^3^; *p* < 0.05 corrected] (Figure 4) was predicted by higher PSQI total scores. No negative connectivity patterns were detected.

In SPSS, *post-hoc* regression analysis revealed diagnostic status did not predict amygdala-postcentral gyrus connectivity *p* = 0.41 (Model 1), and no particular PSQI component score(s) predicted significant functional connectivity (*p* = 0.35) (Model 2).

## Discussion

The present study used a seed-to-whole brain analysis to examine individual differences in subjective sleep quality, indexed with the PSQI (59), on rsFC for bilateral amygdala in individuals with and without an anxiety or depressive disorder. Controlling for symptom severity, left amygdala revealed worse sleep (i.e., higher PSQI total scores) corresponded with increased FC to subgenual anterior cingulate cortex (sgACC) but less FC to posterior cerebellar lobe and superior temporal gyrus. Regarding right amygdala, poorer sleep was linked with increased FC to postcentral gyrus. *Post-hoc* regression analysis did not suggest significant FC results related to diagnostic status, and in general, *post-hoc* regression analysis indicated FC findings were explained by the summation of PSQI component scores. The exception was left amygdala-superior temporal gyrus coupling, which was predicted by the overall sleep quality component, a one-item scale (*very good* to *very bad*) but no other components (e.g., sleep latency, sleep duration, habitual sleep efficiency).

Our hypothesis was partially supported as whole-brain analysis revealed sleep quality modulated amygdala-frontal FC, however, the expected pattern of connectivity extrapolated from case-controlled studies of IPs was not supported. That is, more not less coupling between amygdala and sgACC was predicted by worse sleep when controlling for anxiety and depression symptoms. Findings are generally in line with a sleep deprivation resting-state study that reported increased FC between centromedial amygdala and rostral ACC in healthy individuals relative to rested wakefulness (65) and evidence of increased amygdala-rostral ACC FC in insomnia disorder when compared to generalized anxiety disorder (47). Notably, primate and effective connectivity studies indicate the amygdala has direct connections to sgACC (66, 67), a region implicated in the modulation of emotional behavior (68) and known to be dysfunctional in IPs [e.g., depression; (69)]. Taken together, poor sleep-related increased amygdala-ACC connectivity may reflect a compensatory response or hyperarousal (i.e., aberrant synchronization between linked regions).

Findings expand on a meta-analytic study involving anxiety and mood disorders and at-risk populations (e.g., history of childhood adversity, elevated trait anxiety, behavioral inhibition) that demonstrated aberrant (increased or reduced) FC was isolated to amygdala and ventral portions of the ACC (i.e., pregenual ACC, subgenual ACC) (70). Repeated reports of anomalous FC in amygdala-ACC indicates the sub-circuit plays a role in the general emotional dysregulation observed in the IP continuum. While speculative, our results suggest an aspect of amygdala-sgACC heterogeneity in FC may be explained in part by variance in sleep quality.

Worse sleep quality also corresponded with reduced amygdala-posterior cerebellar lobe connectivity. The cerebellum has extensive anatomical and functional connections with limbic (e.g., amygdala) and prefrontal regions (71, 72), and the posterior cerebellar lobe is involved in emotional control and executive function (e.g., planning, set-shifting, working memory) (73). Although it is unclear whether the region has a direct inhibitory effect on amygdala response or whether the amygdala has a modulatory effect on cerebellar activity, the diminished connectivity associated with higher PSQI total scores suggest worse sleep reduced coupling in a sub-circuit involved in higher-order processes (72). Analogous to these results, less intrinsic coupling between amygdala and superior temporal gyrus was also predicted by poorer sleep quality. There is evidence of a direct connection between amygdala and superior temporal gyrus (66), which is central to auditory processing, language function, and social behavior (74, 75–77). Results indicate more sleep loss may have attenuated amygdala-superior temporal co-processing of external stimuli (e.g., noise related to scanner) or behaviors (e.g., self-monitoring) at rest.

For right amygdala, whole-brain regression results, controlling for symptom severity, revealed worse sleep quality corresponded with increased FC to postcentral gyrus, a sensorimotor region (78). In individuals with primary insomnia, increased resting-state FC between right amygdala and postcentral gyrus relative to healthy controls has been reported (46), therefore, results may reflect the heighted physiological arousal and/or cognitive arousal (e.g., intrusive thoughts) associated with problematic sleep (79–83).

When exploring PSQI components, the only item that significantly predicted significant FC was the sleep quality question, which has a similar inference to the PSQI total score. Altogether, more circumscribed components such as sleep latency, sleep duration, habitual sleep efficiency, or daytime dysfunction did not relate to whole-brain regression findings. Thus, whole-brain results were largely driven by individual differences in global sleep quality as opposed to a particular aspect of self-perceived sleep.

Overall, the evaluation of naturalistic sleep quality on resting-state amygdala connectivity is a novel area of study, therefore, it is important to consider that data from sleep deprivation studies involving healthy participants and case-controlled studies of insomnia disorder may not extend to our cohort who vary considerably in sleep quality and on average, experience less severe or less acute sleep loss. For example, sleep deprivation in laboratory settings alters functional connectivity patterns (84) and weakens the modular functional organization of resting-state networks, including the salience network which encompasses the amygdala (85, 86) resulting in a more random network (87). Even though moderate levels of insufficient sleep, when chronic, impairs neurobehavioral functions (88), it is unclear if more modest sleep loss yields similar effects on network properties. Alternatively, in a departure from the modular model, a contemporary formulation of emotion asserts multiple overlapping networks and coordinated activity in large-scale cortical-subcortical systems underlie emotional states (89). The inference is the positive and negative FC predicted by sleep quality in our study modulates a broad interconnected system of which the amygdala circuit is an important hub rather than impacting discrete core amygdala functions (e.g., generation of affect) (89). Thus, further study is needed to understand the intersection of sleep on intrinsic networks in the IP spectrum.

This study is not without important limitations. First, there was an unequal distribution of patients and healthy participants. Moreover, among patients, more individuals had a primary anxiety disorder than major depressive disorder. Therefore, even though diagnostic status did not appear to significantly contribute to whole-brain findings, the majority of participants were anxious. Second, the patient cohort was highly comorbid, therefore, we cannot disambiguate the contribution of anxiety and depression symptoms on findings. Third, the cross-sectional, correlational nature of this study precludes an ability to make causal inferences. Fourth, we did not screen for sleep disorders, therefore, it is not known whether a portion of participants, including healthy individuals, experienced clinical sleep dysfunction. Fifth, there was no direct manipulation of sleep therefore, results rely on indirect estimates of sleep quality. Sixth, the PSQI, not unlike other self-report measures, is subject to inaccuracy; results may not generalize to objective measures of sleep (e.g., actigraphy).

In conclusion, this is the first study we are aware of to show self-perceived sleep quality modulates amygdala rsFC in individuals with and without clinical anxiety or depression. Several lines of research signify mechanisms of emotion dysregulation and problematic sleep are transdiagnostic and inter-related to varying degrees. Our findings indicate individual differences in sleep quality tracks brain pathways involved in cognitive-emotion functions commonly implicated in the neurobiology of IPs. Therefore, the clinical relevance of identifying transdiagnostic phenotypes may be improved by incorporating sleep measures.

## Author contributions

HK contributed to the study design, interpretation of results, and writing of manuscript. BH analyzed the data. KLP contributed to the interpretation of results and writing of manuscript.

### Conflict of interest statement

The authors declare that the research was conducted in the absence of any commercial or financial relationships that could be construed as a potential conflict of interest.
